# Motivations underlying co-use of benzodiazepines and opioids in the UK: a qualitative study

**DOI:** 10.1186/s12954-025-01312-x

**Published:** 2025-09-29

**Authors:** Gabriele Vojt, Hannah E. Family, Hannah Poulter, Chris P. Bailey, Damiana Cavallo, Ana Paula Abdala Sheikh, Sara Karimi, Nick Booth, Peter Da Silva, Louise Aitken, Samantha Stewart, Matthew Hickman, Graeme Henderson, Jennifer Scott, Joanna M. Kesten

**Affiliations:** 1 National Institute for Health and Care Research (NIHR) Health Protection Research Unit in Behavioural Science and Evaluation, Bristol Medical School, University of Bristol, Bristol, BS8 2BN UK; 2 Population Health Sciences, Bristol Medical School, University of Bristol, Bristol, BS8 2BN UK; 3 NIHR Applied Research Collaboration (ARC) West, 9th Floor, Whitefriars, Bristol, BS1 2NT UK; 4 School of Social Sciences, Humanities and Law, Teesside University, Middlesbrough, TS1 3BX UK; 5 Department for Life Sciences, Centre for Therapeutic Innovation, University of Bath, Bath, BA2 7AY UK; 6 School of Physiology, Pharmacology & Neuroscience, Faculty of Life Sciences, University of Bristol, Bristol, BS8 1TD UK; 7 Developing Health and Independence, Brunswick Court, Brunswick Square, Bristol, BS2 8PE UK; 8 Recovery Connections, 112 - 114 Marton Road, Middlesbrough, TS1 2DY UK; 9 Scottish Drugs Forum, 91 Mitchell Street, Glasgow, G1 3LN UK; 10 Bristol Drugs Project, 11 Brunswick Square, Bristol, BS2 8PE UK; 11 Centre for Academic Primary Care, Bristol Medical School, University of Bristol, Bristol, BS8 2PS UK

**Keywords:** Substance use, Co-use, Benzodiazepines, Opioids, Overdose, Qualitative, Motivations, Harm reduction

## Abstract

**Background:**

Drug-related deaths have substantially increased over the past decade in the UK, particularly in Scotland. Co-use of opioids and benzodiazepines (prescribed and/or illicit) is contributing to rising mortality. This study identified motivations in people’s co-use with the aim of informing prescribing and harm reduction interventions to address drug-related deaths.

**Methods:**

We interviewed 48 people who co-use opioids and benzodiazepines and/or z-drugs (zopiclone and zolpidem) in Glasgow (*n* = 28), Teesside (*n* = 10) and Bristol (*n* = 10). Most participants self-identified as male (*n* = 37, 77%), white (*n* = 45, 94%) and had a mean age of 43 years (range: 25–61 years). The majority reported at least one overdose experience, and poor mental health including trauma. Interviews were semi-structured, conducted by an academic and/or peer researcher, and analysed using reflexive thematic analysis.

**Results:**

Participants’ motivations for co-using mapped onto two interlinked meta-themes: (1) Functional motivations included co-using to augment drug effects, self-medicate or help to generate income. (2) Experiential motivations described participants’ desires to achieve a ‘buzz’ (feeling energised), ‘glow’ (feeling comforted), ‘oblivion’ (escaping trauma and adversity), and ‘gouching’ (physical and mental sensations of ebbing in and out of glow and oblivion). Functional and experiential motivations were dynamic, interrelated and often co-occurred.

**Conclusions:**

The importance of assessing motivations to co-use should be routinely recognised as part of harm reduction and medication assisted treatments to reduce mortality risk.

## Introduction

The co-use of benzodiazepines and/or z-drugs (zopiclone and zolpidem) with opioids is associated with increased risk of drug-related-deaths (DRDs) globally [1]. In Scotland, most DRDs involve opioids (82%) and benzodiazepines (70%) [2]. Co-use is defined as the consumption of two substances, either simultaneously or sequentially, resulting in interacting effects determined by duration of drug action [3]. Co-use of benzodiazepines and opioids (referred to as co-use hereafter), especially when consumed within a wider polysubstance use pattern (e.g., combined with stimulants such as cocaine, and alcohol) are acute risk factors in opioid toxicity, i.e., fatal and non-fatal overdoses. Both opioids and benzodiazepines interact with the brain by binding to different receptors acting on pathways which impact the same outcome, e.g., causing experiences such as euphoria and sedation [4]. Z-drugs act at the same receptors as benzodiazepines and while similar in effect, they are chemically different [5]. The receptors affected by opioids, benzodiazepines and z-drugs impact on respiratory neurons; they slow down breathing. When co-using, benzodiazepines and/or z-drugs may synergistically or individually increase the anti-respiratory effect of opioids, making an overdose more likely. However, the precise neuropharmacological mechanisms underpinning an overdose through co-use are not clear [6].

Treatments to date are focused on single-substance use, typically opioids via prescribing opioid antagonist treatment (OAT) [7]. Interventions for co- and polysubstance use are urgently required to prevent DRDs and improve the support available to people who co-use. One approach to addressing co-use is co-prescribing OAT and benzodiazepines. However, this alone is unlikely to be sufficient as co-prescribing is associated with increased mortality [8] and because co- and polydrug use do not occur in a vacuum. For example, co-use and increased risk of DRDs are related to lower socioeconomic status, poverty [2], low rates of healthcare and treatment access, poor engagement [9] and retention [10], poor physical [11] and mental health [2, 12]. Guidance from Scotland (Medication Assisted Treatment (MAT) standards [13]) suggests that understanding a person’s needs to (co-)use drugs is key in optimising treatment and service delivery. For example, the MAT guidance for benzodiazepine harm reduction emphasises the importance of staff’s ‘understanding [of] presenting issues, predisposing, precipitating and perpetuating factors’ underpinning drug use [14]. In other words, staff are asked to do a psychological formulation. This means they are encouraged to work with service users to come to a shared understanding of the service user’s needs in terms of their previous and current life situation, their coping styles, their experiences and what these mean to the service user [15]. This is reinforced by the UK clinical management guidance on working with people who use drugs [16]. Understanding individuals’ motivations to co-use contributes to aligning harm reduction interventions and treatment with service users’ goals thereby retaining the person in services, creating personal meaning [17] and supporting autonomy [18].

Theoretically, motivations to use alcohol and other substances are conceptualised as interactions of people’s expectations, their learned history and traits (e.g., responses to substance use, past reinforcements) and current contextual factors (e.g., affect, availability, coping skills and physical setting) [19]. The literature on motivations specifically underlying co-use suggests a broad dichotomous framework consisting of (1) self-therapeutic (removing negative emotional states such as anxiety) and (2) hedonic (or seeking euphoria) motivations [20, 21]. However, these findings are anchored in quantitative research designs. A qualitative and mixed methods review of motivations for polydrug use, across different populations, reported eight motivational patterns [22]. Drugs were used sequentially to (a) alleviate withdrawal, and (b) prolong euphoria (‘being high’). Drugs were used simultaneously to (c) balance the effects of drugs, (d) counteract the effects of drugs, (e) enhance euphoria, (f) reduce overall drug use and associated harms (especially from alcohol use), and (g) mimic the effects of substances. When studies did not specify the temporal sequence of polysubstance use, this was typically related to motivations to (h) self-medicate (in relation to pain). In summary, the existing qualitative literature tends to report motivations in functional terms (i.e., self-therapeutic) with less written about hedonic motivations. Findings predominantly reflect a US and Canadian context, with evidence accumulated between 2010 and 2015 and without a focused ‘benzodiazepine-opioid’ lens.

We conducted a qualitative study with people who co-use as part of a wider research project examining the interactions between benzodiazepines, z-drugs and opioids which may increase the likelihood of a fatal overdose. While the laboratory studies aim to specify the pharmacological interactions of opioid and benzodiazepine/z-drug consumption, the qualitative study presented here provides an ecological and frontline context for how and why people co-use. In this paper, we explore self-reported motivations to co-use benzodiazepines/z-drugs and opioids to inform harm reduction and intervention designs to prevent DRDs. We also report on consumption patterns of co-use [23] and perceptions of co-use and overdose risks and prevention [24] separately. We included z-drugs because they are similar to benzodiazepines, and people who use benzodiazepines tend not to use z-drugs, and vice versa [25]. While z-drugs are typically associated with lower DRD rates, there is nonetheless an increased risk of mortality in high-risk populations such as those co- and polydrug using [26].

## Methods

### Study design

We conducted a qualitative study with people who co-use benzodiazepines/z-drugs and opioids in three diverse cities in the UK, i.e., Glasgow (Scotland), Bristol (South-West England); and Teesside (North-East England). Further details, including reflexivity and rationale for settings, are reported elsewhere [23]).

## Participants

We interviewed 48 people with current and recent (in the past 6 months) co-use of benzodiazepines/z-drugs and opioids, with 28 interviews in Glasgow, ten in Teesside and ten in Bristol. Table 1 summarises demographics for the total sample and by study location.

**Table 1:** Participant demographics for the total sample and per study location

		Glasgow (*n* = 28)	Bristol (*n* = 10)	Teesside (*n* = 10)	Total (*n* = 48)
Gender	Male	21 (75.0%)	7 (70.0%)	9 (90.0%)	37 (77.1%)
	Female	7 (25.0%)	3 (30.0%)	1 (10.0%)	11 (21.9%)
Mean age (SD)		42.2 (9.7)	46.4 (6.2)	40.7 (7.3)	42.8 (8.7)
Age range		25–61	41–61	30–50	25–61
Ethnicity	White British	3 (10.7%)	5 (50.0%)	9 (90.0%)	17 (35.4%)
	White English	–	1 (10.0%)	–	1 (2.1%)
	White Scottish	25 (89.3%)	--	1 (10.0%)	26 (54.2%)
	Black British	–	1 (10.0%)	–	1 (2.1%)
	White African	–	1 (10.0%)	–	1 (2.1%)
	Other (Norse)	–	1 (10.0%)	–	1 (2.1%)
	Declined to answer	–	1 (10.0%)	–	1 (2.1%)
Housing	Home/houseless	10 (35.7%)	4 (40.0%)	3 (30.0%)	17 (35.4%)
	Supported accommodation	2 (7.1%)	–	3 (30.0%)	5 (10.4%)
	Rented/ own accommodation	16 (57.2%)	6 (60.0%)	4 (40.0%)	26 (54.2%)
Alcohol (self-defined)	Heavy	8 (28.6%)	2 (20.0%)	2 (20.0%)	12 (25.0%)
	Moderate	5 (17.9%)	3 (30.0%)	2 (20.0%)	10 (20.8%)
	Minimal	7 (25.0%)	2 (20.0%)	2 (20.0%)	11 (22.9%)
	None	6 (21.4%)	3 (30.0%)	4 (40.0%)	13 (27.1%)
	Undisclosed	2 (7.1%)	--	--	2 (4.2%)
Non-fatal overdose experience	Self-reported	21 (75.0%)	7 (70.0%)	10 (100.0%)	38 (79.2%)
Poor mental health	Diagnosed and self-reported	28 (100.0%)	9 (90.0%)	8 (80.0%)	45 (93.8%)

## Procedure

### Recruitment

We recruited from drug treatment and harm reduction services, homelessness outreach, residential crisis and stabilisation services, and support groups including a women’s only group. Gatekeepers (e.g., staff working in study settings) supported the research team with access, identification and recruitment of research participants with a range of co-use experiences using information sheets and posters. People were eligible to participate if they were aged 18 + years, able to converse in English and had co-used benzodiazepines or z-drugs and opioids in the past six months. Interested individuals either contacted the academic researcher directly or liaised via the gatekeepers. Accordingly, researchers arranged interviews with potential participants or through gatekeepers. Opportunistic recruitment also took place with researchers regularly visiting services. Interviews typically took place in secure and staffed settings; on two occasions interviews were conducted in communal settings (e.g., a café) as per participant preference. All interviewees received £10, in cash or voucher depending on the relevant organisation’s policy, as a token of appreciation.

## Data collection

Individual interviews were conducted by an academic researcher (GV, HF, HP) only or co-conducted with a trained local peer researcher (*n* = 18 interviews) depending on availability and the participants and local setting’s preference. Interviews took place between November 2022 and September 2023 in person (*n* = 47) or by telephone (*n* = 1). Interviews lasted a mean of 50 min, ranging from 20 to 103 min. The decision to cease collecting data was informed by the principles of information power [27] assessing the data collected in relation to the study aim, sample specificity (participant characteristics relating to the phenomenon under study), quality and depth of the data, and planned analyses. All interviewees provided written informed consent. Peer researchers and/or the peer research organisation received payment for their time. Interviews were audio recorded, transcribed (intelligent verbatim) and anonymised.

### Patient and public involvement and engagement (PPIE)

Collaborators including service users were involved in all stages of the research development and design. Via recruitment posters, we engaged with a small group of PPIE (*n* = 2 women and *n* = 1 man) who co-use in a Bristol drug treatment service. We conducted an informal group session (using a focus group topic guide) to explore the relevance of our study, the content of the drafted topic guide, and the design, content and language used in the recruitment materials. For example, our PPIE group suggested to incorporate demographic questions into the interview rather than hand out questionnaires, and they highlighted the sensitivities around asking people about overdose experiences. With our peer researchers, we further adapted topic guides in line with local terminology and drug culture in each research location. We also discussed interview content with peer researchers after interview sessions, (a) to debrief but also (b) to exchange interpretations of findings. In this way, our interpretations of data were already informed by lived and living experience when we reality-checked our main findings at two online workshops (in Scotland and England) including people with lived experience. PPIE members here helped us to re-contextualise the importance and relevance of findings for people who co-use.

## Conceptual frameworks

**Fig. 1:**
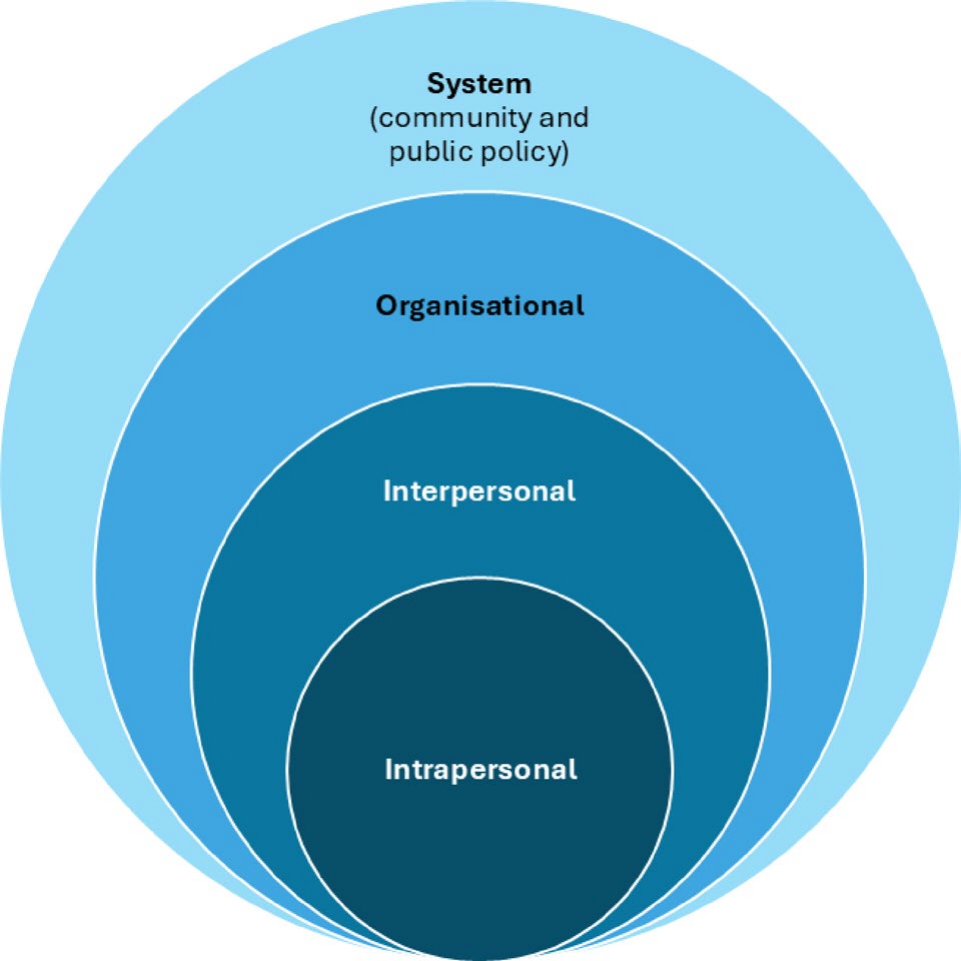
The socioecological framework adapted to health promotion impacts Adapted from McLeroy et al. [28]

Through the socioecological framework lens [28], we developed the topic guide, considered the motivations and wider characteristics of co-using drugs within the context of sociological, cultural, economic structures (Fig. 1).

## Topic guide

The topic guide focussed on the interviewee’s experience of co-using benzodiazepines/z-drugs along with opioids; motivations and patterns of co-use; the role of different types of benzodiazepines and opioids in non-fatal overdose experiences; how the risks of co-use were managed and the characteristics of valuable interventions (see [23] for the topic guide). Interviews also captured sociodemographic characteristics, drug treatment history, mental and physical health conditions.

### Data analysis

All transcripts were analysed inductively and deductively using reflexive thematic analysis [29] in NVivo and on paper. We took a data driven approach following Braun and Clarke’s six-phased method: (1) all anonymised interview transcripts were read and re-read to aid data familiarisation, (2) we open-coded transcripts, guided by participants’ meanings associated with motivations. Next, (3) we collated these codes into themes, (4) we reviewed the generated themes deductively, using existing literature to (5) define and name them and finally (6) write the report. Data analysis was led by a team of researchers (GV, HF, HP); all themes and findings were discussed and refined with senior researchers (GH, JK, JS), and with the wider research team (CB, DC, AS, MH).

### Ethics approval

Ethical approval for this study was obtained from the Faculty of Health Sciences Committee for Research Ethics, University of Bristol (ref 11906).

## Results

**Fig. 2:**
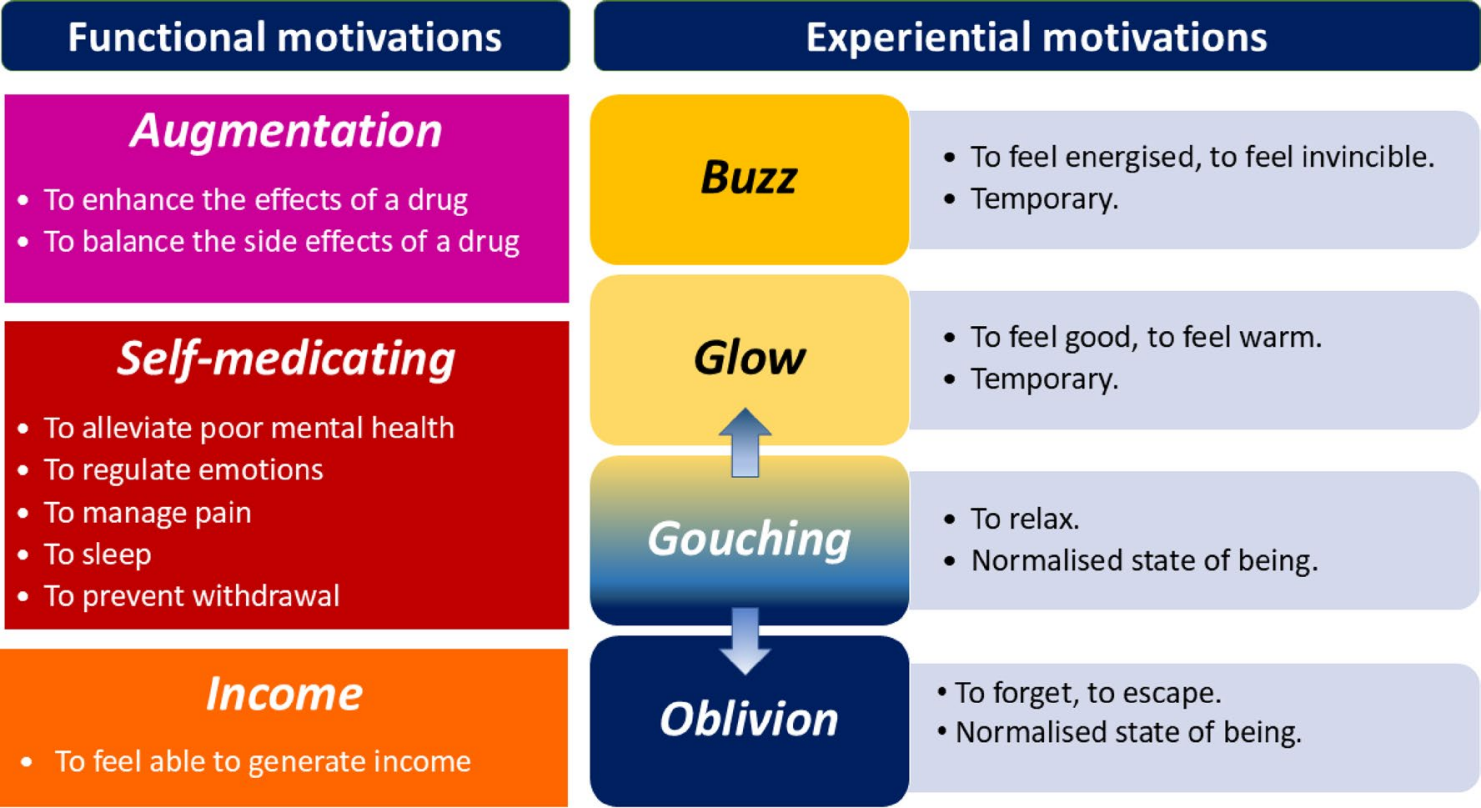
Functional and experiential motivations of co-using benzodiazepines/z-drugs and opioids

We identified motivations to co-use benzodiazepines or z-drugs with opioids, mapping onto two broad interlinked meta-themes; ‘functional motivations’ and ‘experiential motivations’, with associated themes and subthemes. Figure 2 outlines the interconnected meta-themes, themes and subthemes when co-using benzodiazepines/z-drugs and opioids.

Among our sample, co-use of benzodiazepines/z-drugs and opioids ranged from exclusively focussing on benzodiazepines or z-drugs with opioids to co-using within the context of wider polydrug and alcohol use. Here, participants emphasised one or a set of preferred drugs, often benzodiazepines, z-drugs, heroin or crack cocaine. Participants’ narratives included intentional and accidental, non-planned co-use, and reflected a range of self-monitored, restricted vs. intense and binge-like co-use patterns [23]. Many interviewees were uncertain about the actual active ingredients in the substances they used, which prevented linking specific drugs to specific experiences with certainty [9].

### Functional motivations

There were three interconnected themes in participants’ functional motivations; these were *‘augmentation’* – to enhance the psychoactive effects of drugs and/or to counterbalance unwanted side effects from other drugs, *‘self-medicating’* – to alleviate poor mental and/or physical health, and *‘income’* – to feel able to generate income. With input from our peer researchers and from PPIE, we established that functional motivations were the primary drivers for co- and polysubstance use, but also that motivations to co-use could change rapidly depending on situation, mental state, expectations, quality and onset of co-use drugs.

#### Augmentation

Most participants described augmenting, i.e., enhancing the effect (‘hit’ or ‘dunt’) of benzodiazepines or z-drugs with opioids, typically heroin, methadone, oxycodone and codeine. Participants explained that the tolerance to benzodiazepines/z-drugs increases rapidly, therefore co- or polydrug use was often described as essential to augment benzodiazepine-effects to meet expectations and needs.

*‘If I was going out to get the both of them* [heroin and street Valium] *I would take some of the street Valium. I would probably take 25 before I went home.* […] *I would take them and then I would go home and smoke some heroin a bit at a time and bring them* [street Valium] *on.*’ (P13, male, Glasgow).

Benzodiazepines/ z-drugs were also co-used to counterbalance the side effects of other drugs, e.g., to ‘come down’ from cocaine. While those on maintenance prescription of Buvidal^®^ (buprenorphine slow-release injection) tended to poly-use with benzodiazepines/ z-drugs, they did not consider this as co-use with opioids per se, but rather as separate drug-using behaviours that operated in parallel.

#### Self-medicating

Here, participants contextualised their co-use as part of being able to function on a daily basis. Subthemes centre around motivations *‘to alleviate poor mental health’* (e.g., diagnosed anxiety, depression, post-traumatic stress disorder and trauma (particularly among women)), *‘to regulate emotions’* (e.g., stress, social anxiety, daily worries), *‘to induce and enable sleep’*, *‘to manage pain’*, and *‘to prevent withdrawal’*.

Participants often co-used within the context of poor mental health support, insufficient or absent benzodiazepines prescribing and experiences of stigma when asking for support and increases in prescriptions. As a consequence, illicit benzodiazepines in particular were used *‘to alleviate poor mental health’* and *‘to regulate emotions’*. The following participant focuses on the effects of benzodiazepine (on top of Buvidal) when self-medicating with the aim of feeling ‘normal’ as opposed to mentally unwell (e.g., paranoid).

‘[Benzos] *make me feel good* […] *and make me feel calm*,* make me feel lucid*,* make me feel normal in a sense*,* you know what I mean.’* [Can you say a wee bit more, what does it feel like to be normal? ] *‘It just feels calm*,* less paranoid*,* feeling like you’re able to*,* I don’t know*,* just actually step out the door in a sense*,* know what I mean. Usually*,* I just feel like sitting in my room all day*,* depressed* […] *but when I take my benzos I can go out and I can do things. It’s like I’ve had to come for blood tests today*,* I’ve did that*,* know what I mean.’* (P34, male, Glasgow).

Co-use to self-medicate also occurred within a wider context of anxieties, worries and dealing with actual and anticipated life challenges. For example, this participant described co-using in response to feeling overwhelmed by the possibility of losing her supported accommodation, especially because she had experienced homelessness before. The lack of stable housing meant that she was ‘careful’ how she behaved and engaged with housing rules and co-used to block anxieties and stress.

*I’ve just been depressed*,* like hitting the drinks*,* sitting with my pals just trying to kind of block it* [possibility of being made homeless] *out. I started like being back with old friends I used to jump about with when I was using and I had kind of cut that off and moved on but then just filled with anxiety and think I’m going to be homeless again*,* just the anticipation I’ve been taking street Valium* [with heroin] *and sitting with my pals and using again.’* (P39, female, Glasgow).

Poor mental health and trauma experiences were related to the subtheme *‘to induce and enable sleep’* as participants outlined co-use to subdue racing thoughts, primarily achieved by using benzodiazepines and/or z-drugs.

*‘I think I use drugs not to feel good off them but to try and keep it under control*,* my mental health. Basically*,* to block it out because it’s just a daily burden*,* every day.* […] *Your mind races on a night and you can’t stop it. And your body’s tensed up*,* so the Zopiclone is to knock me out and the* [street] *diazepam is to relax me.’* (P25, male, Teesside).

Some participants described co-using specifically ‘*to manage pain’*. This participant described being on prescribed morphine and Valium to manage physical pain and discomfort. When his morphine and Valium prescription was reduced and in the absence of required professional support, he self-medicated by obtaining street opioids and benzodiazepines (e.g., OxyNorm and pregabalin) on top of his prescriptions.

*‘I was on 800 of zomorph* [morphine] *a day*,* so I’ve come down to 110*,* but I started on 800mls* [milligrams] *a day and two bottles of Oramorph* [morphine] *a week. Then I came down to* [area], *they reduced me straight down to 25mgs of Valium from 100. I had about 30 mini-strokes*,* no joke.* […] *I phone my doctor*,* I said*,* ‘Listen*,* I’m in so much pain. I need something for breakthrough pain*,* like a bottle of oramorph*,* you’ve reduced me too fast. He said*, ‘[Name], *I’m not your–‘ I wish I could have recorded it*,* he goes*,* ‘I’m not your fucking joint dealer*,* go and buy some smack*,*’ and hung up.*’ (P17, male, Bristol).

In the subtheme *‘to prevent withdrawal symptoms’*, the fear or anticipation of potentially withdrawing motivated co-use as much as the actual experience of withdrawal. This then created a cycle of co-use necessitating a regular supply of substances.

*‘There would be really bad withdrawal symptoms that you would get from them* [co-using street Valium and heroin] *so I think all I wanted to do was to get that off as well. It was like a mind thing as well. If you knew you didn’t have them and you knew you had run out it would be a really horrible feeling so you would need to get your heroin and you would need to get your street Valium just to take that edge off it. It was that really horrible feeling you would get from both of them.* […] [If I was withdrawn] *I would basically be feeling absolutely awful. I would be feeling terrible. I would be shaky. I would be just all over the place.’* (P13, male, Glasgow).

While participants tended to describe withdrawal symptoms from benzodiazepines/z-drugs as more severe and debilitating than opioid withdrawal, we cannot determine with certainty in our data whether participants took benzodiazepines/z-drugs specifically to suppress opioid rather than benzodiazepine/z-drug withdrawal.

#### Income

Some participants described co-using *‘to feel able to generate income’*, such as criminal activities, sex work and other forms of paid labour. Often, participants’ narratives highlighted a sense of routinised and normalised co-using procedures as part of working and making money. Co-using to partake in criminal activities (e.g., shoplifting, burglary, selling drugs) served the function of numbing yet energising the person by instilling confidence. Participants also reported that benzodiazepines and z-drugs made them feel invisible and invincible.

*‘Downers* [heroin] *just block things out. Just what you feel* [is] *numb… and I think see*,* that pairs well with Valium*,* like I’m saying I might think I’m alright but other people are seeing me present and I’m mad with it*,* but I’m not thinking I’m mad with it and when I come back and that whole criminal side*,* that shit again*,* when you’re taking Valium you think nobody sees you*,* you think you’re invisible*,* you’ve got that confidence for you*,* you don’t care about anything*,* and I’m back in shops shoplifting and all that.’* (P39, female, Glasgow).

***‘****I used to always get the jail because I used to do robberies. I used to rob post offices and building societies and that*,* and they* [street Valium] *gave me Dutch courage. The Zopiclone and the Valium*,* they’d be for Dutch courage. Aye*,* they make you invincible and good for graft.’* (P1, male, Teesside).

Participants rarely volunteered information specifically on co-using within the context of sex work. Instead, participants – typically females – discussed co-using as a means to do *‘whatever I had to do’* in order to generate enough income to secure further drugs, which then helped them to function in other life areas. Some participants described long-term co-use as a driver to perform professional duties. Often, this was interlinked to people discussing co-use as a means to stay calm and to negate physical symptoms of withdrawal, pain or stress. The following extract exemplifies using illicit benzodiazepines and heroin before engaging in labour jobs, with the focus on heroin as the main drug to function professionally.

‘*There were times when I would get up in the morning and I knew I had the job to go to at ten and I would nip away and get myself something*,* just maybe a little bag of heroin.* […] *I wouldn’t take as many* [street] *benzos but would take a little bit of heroin and that sort of stuff just so I wasn’t shaky. I would do laminate flooring for people and build bits of furniture for them. It wasn’t any major jobs or anything like that but just your handyman jobs that I would do. That got me through that to be honest with you.’* (P13, male, Glasgow).

In summary, across the functional motivations, co-using benzodiazepines/z-drugs and opioids served as a survival mode within a wider context of adverse life events, loss, disadvantage and trauma. Participants’ motivations were dynamic; they fluctuated, co-occurred and were influenced by context, situation, mental health state and the person’s expectations and experiences from co-use. For example, a person might co-use initially to self-medicate. However, if the quality, onset or dosage of obtained drugs did not achieve the desired effect (as expected or needed), then further motivations to co-use could merge with augmentation. Further, subthemes in self-medicating often co-occurred, e.g., co-using to block out poor mental health while also preventing withdrawal and being able to sleep.

### Experiential motivations

Interlinked to functional motivations and often co-occurring, participants described their motivations to co-use in terms of achieving specific bodily and mental experiences. Experiential motivations described the following themes: *‘buzz’* (feeling invincible and energised), *‘glow’* (feeling comforted), feeling *‘gouching’* (physical and mental sensations of ebbing in and out of glow and oblivion) and *‘oblivion’* (forgetting or escaping adversity and trauma). Participants in our sample described experiences of *‘buzz’* and *‘glow’* using interchangeable terminology, e.g., both experiences were referred to as relaxing but also energising, depending on dosage, co- or polysubstance use combinations and situational context [23].

#### Buzz

Those participants who primarily sought to experience a *‘buzz’* described this as a temporary feeling of energy, increased confidence and self-efficacy (a person’s belief in their ability to complete a task or achieve a goal) to the extent that participants believed themselves to be invincible and/or invisible. Seeking this experience was often linked to functional motivations such as engaging in criminal activities, although equally to obtain sufficient energy to perform daily life functions such as doing housework.

*‘Zopiclone*,* they work relatively fast. But the effect they have on me* […] *they give me a bit of a high. If I can’t be bothered to do nothing*,* I take a couple of them*,* get my housework done*,* and then you just sleep. I’m just ready to shower and then sleep*,* but sometimes I don’t get that far.’* (P28, female, Bristol).

#### Glow

When defining *‘glow’*, participants tended to highlight that these positive experiences were benzodiazepine/z-drug-driven (depending on study location, i.e., zopiclone was primarily discussed in Teesside and not at all in Glasgow) and induced physical sensations of warmth, comfort, happiness and connectivity.

[What does glow feel like? ]*‘The feeling is a very overwhelming feeling of warmth*,* a feeling of there’s not a person who wouldn’t accept you*,* it makes me feel good.’* (P48, male, Glasgow).

Some participants contextualised *‘glow’* by referring to media presentations (e.g., ‘Ready Brek’- a 1980’s porridge advert depicting a person with an orange glow while looking happy and confident) or describing personal experiences of safety (e.g., when visiting their grandparents and being cared for).

‘[Nitrazepam] *just made me feel better. Made me sleep. Made me calm. Made me not sweat. Yeah*,* makes me feel nice.’* [We’ve heard lots of people have said a kind of warm glow type feeling.] *‘Yeah. The ready brek glow I call it.’* (P6, female, Bristol).

[The glow, can you describe it for me? ] *‘It’s like you’re nowhere for three days and going up to your grannie's*,* and you can smell that home baking*,* and you knew there were a big steak pie or something homemade maybe. That was the only time I really got a good meal*,* I went to my grannie's.’* (P15, male, Glasgow).

#### Gouching

*‘Gouching’* was described a slow pattern of ebbing in and out of deep relaxation with intermittent sensations of ‘buzz’ or ‘glow’. Opioids were used as the foundation to induce deep relaxation to the extent of being immobile and oblivious to one’s surroundings, with repeated benzodiazepine consumption to ‘peak’ or ‘wake up’ in a glow-like elation and then submerge back into a state of relaxation. These two participants described the changes in experiencing benzodiazepines when re-dosing while gouching.

*‘When you gouch*,* your body’s jelly. Your body’s just like jelly. Sometimes you cannae even move.* […] [After using heroin] *you just take about 25 of the Valium*,* shove them in my mouth and chew them*,* and as I chew*,* I’ll eat a bit of cake*,* drink coffee*,* then we’ll take another 25 and then another 25 as long as we feel great*,* we feel fine. But we don’t sleep. We gouch*,* wake up; gouch*,* we wake up.’* (P21, female, Glasgow).

*‘It’s* [gouching] *like a peak*,* it’s like it peaks*,* it goes down then peaks again and then goes down and then peaks again and then goes down.’* (P43, male, Glasgow).

#### Oblivion

Interconnected to ‘gouching’, we identified a motivational theme *‘oblivion’*. Those who co-used to experience oblivion often sought a space where they may exist but not necessarily experience or feel. In this sense, oblivion was typically mentioned within the context of self-medicating to alleviate and escape overwhelmingly negative experiences (e.g., life problems), trauma and overall poor mental health. The following quote conceptualises oblivion within the context of a binge-using pattern; while the participant outlines consuming alcohol, prescribed methadone and diazepam, this occurred within a wider context of polydrug use (e.g., street diazepam, cocaine, pregabalin) depending on financial resources.

*‘They* [benzodiazepines] *took me into oblivion*,* they took me away from all my problems*,* I didn’t need to worry about anything*,* nothing bothered me*,* it was just like excuse the line but it was just basically*,* “Fuck everybody*,* I can just do what I want and that’s it.” That’s why I liked it because I could just take them and it was like there was no worries*,* there was no hassle*,* there was no wondering who was going to phone me today or who’s going to be moaning at me*,* work isn’t going to be chasing me up all of that. It just went out the window plus into the bargain it sort of gave me confidence*,* it gave me false confidence*,* false sort of bravado*,* all that sort of stuff* […] *I was that much in oblivion with drugs and alcohol combined on top of 100 and odd methadone prescription a methadone prescription a diazepam prescription.’* (P10, male, Glasgow).

Similar to functional motivations, experiential motivations fluctuated, co-occurred and at times merged with one another. There was a sense that motivations could be singular or primary (such as P10 who sought to achieve oblivion to escape problems) where any add-on effects like an initial buzz (from benzodiazepines) were seen as a pleasant bonus but not the primary driver. In contrast, others described their primary motivation as two-fold, i.e., equally divided into seeking to feel good and elated (buzz) followed by numbness, which was related to gouching and oblivion.

[Earlier on you were talking about the buzz, was that with the street tablets? ] *‘Yeah*,* you have a self-confidence you think you’re invisible*,* you think you’re straight as a die and you’re not. If I was to get a batch of Valium off the street and they were good*,* I’d be like I’d get a gouch*,* too.* [What does that feel like? ] *It just numbs all the feelings like just the abuse growing up*,* trauma on top of trauma on top of trauma. It just numbs it*,* and it makes you not feel anything.’* (P20, female, Glasgow).

## Discussion

In this paper, we identified interrelated and dynamic functional and experiential motivations for co-using benzodiazepines/z-drugs and opioids. Functional motivations included a person’s objective to function on a daily basis. Here, co-use served to ‘self-medicate’, e.g., alleviate/prevent withdrawal and poor mental health, but also to ‘enable income generation’ and to enhance or counterbalance the effects of other drugs (‘augmentation’). Experiential motivations described how co-use felt to the person, cognitively and physically, and ranged from feeling energised (‘buzz’), happy (‘glow’) to deeply relaxed (‘gouching’) and numb (‘oblivion’). Functional themes and subthemes were distinct (i.e., based on participants’ narratives about the main motivation to co-use), however, they could co-occur simultaneously and sequentially. For example, a person might be co-using primarily to block out anxieties and stress (‘to alleviate mental health’ and ‘to regulate emotions’) but at the same time, they may also engage in co-use to augment a desired effect (e.g., to increase euphoria or manage side effects). Likewise, functional and experiential motivations were discussed separately, but could co-occur. For example, a person might co-use to get the courage or energy to make money (‘income’) but co-uses in such a way that there is a buzz or glow experience as a pleasurable, temporary bonus. Motivations, alongside available resources and access to drugs, influenced the timing, frequency and dosing. For example, when the motivation was to gouch, co-use tended to be prolonged with large (re)doses consumed. Seeking oblivion tended to be related to binge-like or extended consumption patterns to remain in a state of disconnection. When the aim was to function, co-use tended to be more structured and controlled, especially to attend meetings and carry out tasks (e.g., go to work, shop for groceries, collect medication) [23].

### Comparison with existing research knowledge

Our findings extend the existing literature on motivations to co- and polydrug use at a dichotomous level (self-therapeutic to ‘functional’ vs. hedonistic to ‘experiential’) [20] and at a granular level [22, 30]. The experiences ‘glow’, ‘oblivion’ and ‘buzz’ sought when co-using build on existing descriptions such as ‘a little glow all round me’ [31], seeking a ‘blackout’ [21] and wanting to feel ‘euphoric’ [32]. We found that while there were nuances and differences between motivations, there were also overlaps depending on participants’ understandings, definitions and personal meaning-making of motivations. For example, the experiential motivations of ‘buzz’ and ‘glow’ were clearly distinguishable based on participants’ accounts, and yet the terms were used interchangeably. Likewise, there were clear differences between ‘gouching’ and ‘oblivion’, and yet some participants combined these terms into one construct. This is in line with previous research considering motivations to co-use in a dichotomous framework. Co-use enabling people to generate income via criminal activities and sex work is well documented [30]. However, our findings further evidence that co-use may also enable people to function physically and mentally in labour jobs. Central to many of the functional motivations for co-use is self-treatment in line with drugs’ medicinal function [22, 30]. For example, participants spoke of seeking medication for physical and mental health conditions suggesting unmet treatment needs. Reported challenges in accessing prescribed medications are associated with individuals attempting to address such unmet needs with illicit medications [31]. That is, some participants in our study sought out street benzodiazepines to numb the impact of historical and ongoing disadvantage including sexual, violent and psychological trauma, self-reported poor mental health including diagnoses, and challenging life experiences from early childhood to adulthood (e.g., homelessness, poor physical health). Consequently, we found that motivations in our sample were typically contextualised within a relief (e.g., self-medicating) rather than a reward (e.g., euphoria) paradigm [32]. However, this is not to say that participants did not enjoy co-use.

### Implications

In terms of general implications, motivations to co-use should not be considered in discreet categories, but rather within a wider framework to better contextualise and understand the person, and to help the person understand themselves. The key is to understand that there is not a linear relationship between motivation to co-use and ‘doing the co-use’, but rather a complex, interconnected web with primary motivation(s) at the core, but these – very much in line with motivational theory – can change according to situation, mental state, expectations and the quality, dosage and onset of drugs being used. Our findings have multiple implications for therapeutic interventions highlighting the need for pathways to screen, diagnose and adequately treat mental health symptoms (e.g., anxiety) and to address the experiential and functional effects of benzodiazepines used by people co-using opioids [20, 33]. Importantly, understanding motivations for co- and polysubstance use should be routinely assessed and incorporated into clinical decision making on (co-) prescribing, drug treatment interventions and harm reduction. Using the socioecological framework, implications can be mapped at the intrapersonal, interpersonal, organisational and system level [28].

At the **intrapersonal level**, and within the context of psychological formulation (i.e., understanding how, why, when and under what circumstances a person came to co-use in the past, and what sort of factors might keep co-use going), our findings highlight the need to enable, empower and support people’s own sense making of their co-use motivations. This should occur within a broader understanding of core beliefs and values, sense of self, coping style, emotional regulation skills as well as one’s hopes and desires. Fostering such insights and reflections in service users is key to empowering informed, personal choices, and co-designing and agreeing on meaningful, realistic and flexible treatment plans and individual harm reduction strategies. This aligns with the personalised care approach [34] and has been used in long term physical and mental health conditions [35]. To help staff support service users, routinised and meaningful assessments of motivations could be utilised to inform motivational interviewing and enhance clinical and psychosocial assessments. The systematic measurement of motivations for co-use using tools such as an inventory of drug taking situations [36] or a visualised pathway map could underpin shared decision making to (co-)prescribe. Such a situation or context specific inventory/ map aligns with motivational theory. For example, our results demonstrate that benzodiazepine-intake was motivated by both, to get a buzz but also to relax, depending on the situation. The experience and expectation of these sensations were related to co-use patterns, dosage and frequency of benzodiazepine/z-drug use [23].

At the **interpersonal level**, service users’ perceptions and understanding of drug related harms may require upskilling people who co- and polydrug use prophylactically to balance individuals’ motivations against risks of harm. This is particularly important considering that motivations to use drugs are not discreet [22, 30] nor stable [20]. Therefore, prescribers, drug treatment, harm reduction and support staff have the opportunity to help service users to identify the importance, function and priority co-use has on a daily basis, and more generally across the person’s life. Here, harm reduction staff are encouraged to maximise on all opportunities to engage and assess people’s motivations to co-use to integrate this into a structured referral system. For example, if co-use is primarily driven by functional motivations such as self-medicating to suppress poor mental health, then harm reduction staff can signpost and refer on to specialist services including mental health, counselling and/or drug treatment teams. In terms of experiential motivations, if service users describe co- or poly-using primarily to gouch out or to achieve oblivion, then this is likely to indicate a crisis situation where the service user is not necessarily focussed on their safety and wellbeing while using drugs. In the short term, the aim then is to ensure the person is supplied with naloxone, and if available be referred to a supervised healthcare setting such as a safer consumption room. In the long term, medication assisted treatment, peer support and psychosocial interventions are essential. Likewise, prescribers’ decision making will benefit from systematically assessing motivational drivers. For example, if a person describes their motivations to co-use in functional terms, then prescribers could implement split-dosage prescribing tailored to the daily contexts when functional motivations are most prevalent (e.g., early mornings to get out of bed, or later in the day to leave the house to go to the pharmacy). If this is not possible, then prescribers could use motivational understandings to optimise the person’s OAT prescription (dosage or type of OAT) and/or manage potential side effects from other prescribed drugs which the person might try to alleviate via co-use. For example, people who co-use benzodiazepines and methadone are reported to have objectively poor sleep and suffer from insomnia [37]. Prescribers and patients could explore a move from methadone to buprenorphine or consider sleep treatment to circumvent the need to co-use. In this way, realistic, sustainable and individualised harm reduction and intervention plans can be co-produced and implemented in the short- and long-term.

**Organisationally**, a staff culture underpinned by psychologically framed values and ethos could be strengthened. Awareness and an understanding of functional and experiential motivations to co-use should be reinforced. While organisational change in healthcare settings is notoriously challenging [38], an example from the UK, Scotland, highlights that healthcare, drug treatment staff and prescribers have been trained up in psychologically formulating (i.e., co-reflecting with the service user on the factors contributing to co-use) people’s motivations to (co-)use drugs (Traynor, 2024, personal communication). Therefore, staff-wide upskilling to consider people’s behaviour within a motivational and psychological context may be feasible. However, improved economic resources and funding avenues are required to endorse and implement staff training, in particular to prevent staff burnout when faced with the complex realities and reasons of people’s co-use and life experiences [39]. Carlisle and colleagues [40] underline the dilemma staff in drug treatment services face in terms of organisational policies and pressures. On one hand, staff are required to facilitate successful, drug-free discharges, yet with relatively little time to understand the reasons (or motivations) for people’s drug use. Therefore, awareness of our motivational themes and their behavioural impact may support aims to retain service users and help to design responsive care and treatment planning. This in turn may increase intervention effectiveness in the long-term (by being able to update treatment to individual needs and priorities) and reduce potential healthcare and service costs incurred through overdose reactive care and discharge from services [7, 39]. To achieve this, our findings may encourage the establishment of a shared vocabulary between service users and staff, opening up a safe space where potential power differentials can be navigated. A local/regional list of key terms and associated motivations (e.g., ‘oblivion’) could be used to alert staff to probe further in a non-stigmatising, inclusive and open-ended format (e.g., ‘what is wrong with you?’ vs. ‘what has happened to you?’) [41]. This could then inform referral decisions to appropriate treatments, e.g., trauma, mental health counselling or peer-facilitated activities and support groups. In addition, psychosocial support, harm reduction and drug treatment should not be limited to those describing relief motivations (e.g., self-medicating or seeking oblivion or to gouch), but also those seeking reward motivations (e.g., augmentation, in particular to increase feelings of buzz and glow). For example, overdose prevention centres operate within a paradigm of assisting individuals to use drugs (including motivations to enjoy and feel pleasure or euphoria) as much as to do so in a safe way (harm reduction) [32].

At the **system** level, the main implications are three-fold: firstly, existing interventions and practice such as motivational interviewing should be aligned with guidance and psychology approaches to formulation among staff working with people who co- and polydrug use. Secondly, structural support provision to reduce the risk of DRD should be endorsed via drug testing, naloxone availability and overdose prevention centres. Thirdly, the underlying motivation for people to co- or polysubstance use is to function, often in disadvantaged, impoverished and unstable contexts. Increasing people’s ability to function, to have a sense of control and agency in their decision-making and daily life means that contributing key social and environmental factors need to be addressed. Therefore, improved housing, employability, social connectivity, interventions to improve wellbeing and engagement with services remain a key aim [7] yet a system-wide gap.

### Future research

To optimise treatment and interventions, future gender-responsive research on the trauma and mental health needs are required among people who co-use specifically to self-medicate, seek oblivion or to gouch. Further research exploring service provider views on risks and benefits inherent in current clinical practice and harm reduction approaches and experiences of co-prescribing in the context of opioids and benzodiazepines alongside OAT is needed.

#### Strengths and limitations

We focused on a snapshot of people’s self-reported experiences of co-use, which is unlikely to fully account for the dynamic interactions between motivations, situations and interpersonal responses. We did not include quantitative measures or cross-validation of drug strength or dosage. Further, while our sample was reflective of the national demographics of high-risk groups co-using substances in the UK, we failed to recruit from diverse ethnic backgrounds. We focussed on participants in urban inner cities, and therefore our implications are unlikely to transfer easily to people who co-use in rural settings. We did not examine the motivations and impacts for polysubstance use per se, or for drugs other than benzodiazepines/z-drugs and opioids. Strengths include the involvement of local peer co-researchers and input from a multi-disciplinary team (pharmacists, experimental laboratory, third sector, psychology, behavioural sciences, epidemiology). Analytic generalisations were built through rigorous inductive analysis and triangulation across three geographically diverse research sites to develop broad theories, or conceptualisations in relation to the study aims, objectives and research questions. To support the transferability of the research, we involved peer researchers and national expert stakeholders (including third sector, policy makers, academic researchers and public health professionals) to sense check, fine tune and consider the applicability of implications to other settings and populations.

## Conclusion

This study generated an in-depth and nuanced insight highlighting the complexities and relationships between co-use motivations. Our findings confirmed that co-prescribing benzodiazepines and opioids in the absence of psychosocial and structural support is unlikely to meet underlying needs. Understanding motivations is key to providing individually tailored harm reduction, which in turn link theory to practice and underpin effective treatment and patient-centred care. Utilising the understanding of interrelated motivations to co-use should be incorporated into staff training to provide psychosocial support to people in treatment and people benefitting from harm reduction advice and advocacy.

## Data Availability

Data are available on application at the University of Bristol data repository (data.bris). Data access is restricted to bona fide researchers for ethically approved research and subject to approval by the University’s Data Access Committee.
